# Feedback System Analysis of a Multicomponent Intervention on Dyads of Home-Dwelling Persons With Dementia and Their Caregivers: Results From the LIVE@Home.Path Trial

**DOI:** 10.1093/geroni/igae020

**Published:** 2024-02-23

**Authors:** Maarja Vislapuu, Monica Patrascu, Heather Allore, Bettina S Husebo, Egil Kjerstad, Marie H Gedde, Line I Berge

**Affiliations:** Centre for Elderly and Nursing Home Medicine, Department of Global Public Health and Primary Care, University of Bergen, Bergen, Hordaland, Norway; Centre for Elderly and Nursing Home Medicine, Department of Global Public Health and Primary Care, University of Bergen, Bergen, Hordaland, Norway; Complex Systems Laboratory, Department of Automatic Control and Systems Engineering, University Politehnica of Bucharest, Bucharest, Romania; Department of Biostatistics, Yale School of Public Health, New Haven, Connecticut, USA; Centre for Elderly and Nursing Home Medicine, Department of Global Public Health and Primary Care, University of Bergen, Bergen, Hordaland, Norway; Neuro-SysMed Center, Department of Global Public Health and Primary Care, University of Bergen, Bergen, Hordaland, Norway; Department of Health and Social Sciences, NORCE Norwegian Research Centre AS, Bergen, Hordaland, Norway; Centre for Elderly and Nursing Home Medicine, Department of Global Public Health and Primary Care, University of Bergen, Bergen, Hordaland, Norway; Haraldsplass Deaconess Hospital, Bergen, Norway; Centre for Elderly and Nursing Home Medicine, Department of Global Public Health and Primary Care, University of Bergen, Bergen, Hordaland, Norway; NKS Olaviken Gerontopsychiatric Hospital, Askøy, Norway

**Keywords:** Complex intervention, Complex systems, Informal care, Resource utilization, Steady-state error

## Abstract

**Background and Objectives:**

Proper symptom management, informal caregiver support, and service innovation are required to reduce dementia care burden. The objective of this study is to investigate the effect of the multicomponent LIVE (Learning, Innovation, Volunteering, Empowerment) intervention on caregiver experience of the self-perceived care situation, coordinator performance, and informal care time.

**Research Design and Methods:**

We conducted a 24-month multicomponent, stepped-wedge randomized control trial including dyads of people ≥65 years with mild-to-moderate dementia with minimum weekly contact with their informal caregivers in Norway. The intervention was implemented by municipal coordinators over a 6-month period. This study investigates the first 6-month period (September 2019–March 2020) of the trial, due to the coronavirus disease 2019 (COVID-19) pandemic. Primary outcomes are changes in provision of informal care time assessed by Resource Utilization in Dementia Care (RUD) and informal caregiver experience assessed by the Clinical Global Impression of Change (CGIC). We use logistic regression and feedback system analysis to assess the reach of the multicomponent intervention.

**Results:**

A total of 280 dyads were included at baseline, mean age of the person with dementia was 81.8 years, and 62.5% were female. After 6 months, the feedback system analysis reveals that the caregivers randomized to the intervention period reported improved caregiver situation (CGIG-T: intervention 0.63 (*SD* 2.4) vs control −0.43 (*SD* 1.7), *p* < .01), even though informal care time for activities of daily living was not reduced (*p* = .31). Informal caregivers registered a positive change for the Learning, Innovation, and Empowerment components, while no change was found for Volunteer support.

**Discussion and Implications:**

Findings illustrate the usefulness of dementia care coordinators that provide regular follow-up. We also show that complex intervention studies benefit from applying feedback system analysis. Meeting the needs of persons with dementia and their caregivers is a complex process that requires coordinated input from health services and user communities.

**Clinical Trial Registration Number:**

NCT04043364

**Translational Significance:** Complex interventions are necessary to alleviate the unmet needs of informal caregivers for persons with dementia, covering education, assistive technology, volunteer support, advance care planning, and medication review. This research demonstrates that designated dementia care coordinators play a crucial role in informal dementia care by providing regular, systematic follow-up in a primary healthcare context. Feedback system analysis can lead to a deeper understanding of the synergy between coordinators and patient–caregiver dyads and inform policy change to improve the overall care situation for persons with dementia living at home.

The global prevalence of dementia is expected to triple from 57 million to 153 million people by 2050 (Nichols et al., 2022). This growth is changing the expenditures and can overwhelm health and social services, including long-term care worldwide (Velandia et al., 2022; Wimo et al., 2018). Most older adults, as well as persons with dementia, wish to age in place (American Association of Retired Persons, 2021; Fæø et al., 2020; Lord et al., 2016), which is beneficial for the society due to its positive cost-efficacy (Ekman et al., 2021; König et al., 2014). However, this is a mixed-blessing, as home-dwelling persons with dementia pose an extra challenge for informal caregivers who often provide round-the-clock support (Gillespie et al., 2014; Lindeza et al., 2020). A systematic review by Angeles et al. (2021) identifies disease severity, functional level, and behavioral and psychological symptoms in dementia (BPSD) as the most time-consuming factors for increased informal care at home and in nursing homes (Angeles et al., 2021). Several other studies show that co-residing caregivers (e.g., spouses), provide higher volume of care hours than non-co-residing caregivers (e.g., adult–children; Michalowsky et al., 2018; Nakabe et al., 2019; Ydstebø et al., 2020). There is a strong correlation between time devoted to caregiver tasks in older adults with chronic complex conditions and perceived caregiver burden (Lin et al., 2019; Rodríguez‐González et al., 2021; Tooth et al., 2005). Therefore, reducing the caregivers’ provision of care time or the caregiver burden can potentially reduce the risk of adverse health outcomes for the caregivers, as well as prevent or delay institutionalization of the person with dementia. Long illness duration, impaired cognitive function, and the development of BPSD are main drivers of caregiver burden (Rodríguez‐González et al., 2021) leading to emotional and physical distress for the caregiver (Lindeza et al., 2020; Schulz & Sherwood, 2008), and changes in roles and relationships (Brodaty & Donkin, 2009; Lindeza et al., 2020).

To support informal caregivers at home, several interventions have been implemented and tested (e.g., psychoeducation, cognitive behavioral therapy, support groups, counseling, respite care, and care coordination; Walter & Pinquart, 2020; Williams et al., 2019) with small-to-moderate positive effects on caregiver outcomes such as burden, caring abilities, subjective well-being, depressive symptoms, and anxiety (Cheng et al., 2020; Pinquart & Sörensen, 2006; Walter & Pinquart, 2020). Meanwhile, only a few randomized controlled trials (RCT) explore the effect of a multicomponent intervention on resource utilization (hours of care time provided) in this setting. The U.S. multicomponent MIND (Maximizing Independence in Dementia) RCT included persons with dementia and their informal caregivers (*n* = 289 dyads) to obtain tailored services and delay institutionalization (Samus et al., 2014). This trial shows a significant reduction in informal care hours (Tanner et al., 2015), increased use of outpatient mental health visits, and respite opportunities for the MIND intervention group (Amjad et al., 2018).

The Norwegian Dementia Plan 2020 highlights the necessity for a tailored, multicomponent, clinical pathway including user involvement, better information, volunteerism, joint commissioning of care services, and follow-up by dementia care coordinators (The Ministry of Health and Care Services, 2015). A review of national dementia care strategies shows the importance of dementia care coordinators in follow-up as one of the key actions in several countries (e.g., Japan, France, and Ireland; Chow et al., 2018). The British Medical Research Council framework (2021) emphasizes that researchers must consider the complexity of the development, implementation, and evaluation of complex interventions and interactions within the context (Skivington et al., 2021). Complex healthcare interventions can also be considered complex systems, encompassing multiple interacting elements (e.g., humans, services, and policies) and feedback loops, where the subjects receiving the intervention (e.g., patients) send information back to the providers of interventions (e.g., healthcare workers), and in which both parts may interactively modify their aspects of performance (Burton et al., 2018; Clark, 2013; McGill et al., 2021; Seys et al., 2019; Skivington et al., 2021). However, the causal complexity of these caregiving systems cannot be reduced to linear relationships only (Stroud & Larson, 2021), and consequently, the evaluation of such interventions needs feedback system analysis to gain a deeper understanding of their reach and performance (McGill et al., 2021). Feedback system analysis is a method that evaluates the interactions between interdependent systems as a whole and is able to quantify how changes in one part (e.g., adding the coordinator to care as usual) affect the rest (Åström & Murray, 2021; Morrow-Howell et al., 2017).

The LIVE@Home.Path is a 24-month multicomponent, randomized controlled trial including home-dwelling persons with dementia and their informal co-residing and visiting caregivers (dyads) aiming for longer, safer, and more independent living at home (Husebo et al., 2020). LIVE is the acronym for the multicomponent intervention of Learning, Innovation, Volunteerism, and Empowerment, tailored and implemented over 6 months by skilled municipal coordinators. The trial design entails regular communication between coordinators and dyads during the intervention period, which creates a complex interaction between them, as the coordinator adjusts the provided intervention components based on feedback from the dyads. This generates a causal feedback loop, which requires feedback system analysis.

The aim of this study is to investigate the effect of the intervention on informal care time related to activities of daily living (basic and instrumental) and the caregivers’ experiences of the self-perceived care situation after the first 6-month intervention period. We hypothesize that the LIVE intervention:

(1) improves the self-perceived caregiver situation for the overall sample.
(2) reduces informal care time burden by coordinator led tailoring of the services to the dyads needs.

## Method

### Study Design and Setting

**Figure 1.**
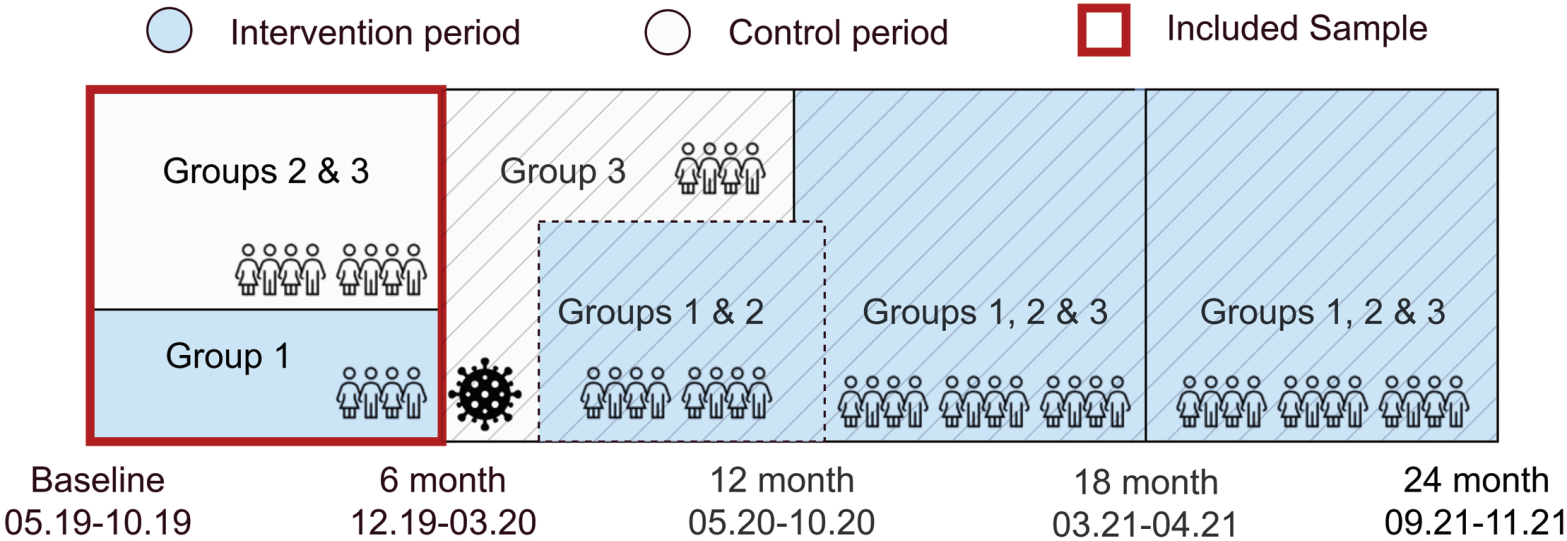
The stepped-wedge study design.

The LIVE@Home.Path study is a 24-month closed-cohort multicenter, stepped-wedge RCT in which the LIVE intervention is implemented in dyads of home-dwelling persons with dementia and their caregivers (Husebo et al., 2020). A stepped-wedge trial is a one-way crossover trial in which several allocation groups are randomized to separate sequences that determine the time point at which each group will switch to the intervention period (Figure 1; Hemming et al., 2015). The design implies that all participants are recruited before randomization, exposed to both the control and the 6-month intervention sequence, and are assessed repeatedly every 6 months. For the 24-month LIVE trial, this yields in total of five cross-sectional data collections at discrete times. Three sequences of intervention periods are implemented during the whole trial, and each sequence includes approximately 1/3 of the recruited participants. Coordinators and dyads remain blinded to allocation status until the sequential rollout. The first 6-month intervention period was completed before the Norwegian government adhered to national coronavirus disease 2019 (COVID-19) restrictions (on March 12, 2020), halting the study protocol (Gedde et al., 2022; Vislapuu et al., 2021). The data collectors were not able to visit the dyads at home, thus the intervention implementation sequence 2 had to be postponed. Thus, in our current study, the analyses are restricted to the period 0–6 months (Figure 1). Additional information on the intervention development, sample size calculations, implementation, and evaluation is presented in the study protocol (Husebo et al., 2020).

### Participants, Recruitment, and Sample

The LIVE trial applies convenience sampling in recruiting dyads from outpatient clinics in specialized dementia care, municipality-based memory teams, and advertisements in the media. Inclusion criteria are persons with a formal diagnosis of dementia, age ≥65 years, Mini-Mental Status Examination (MMSE) score between 15 and 26, and Functional Assessment Staging score between 3 and 7. We consider caregivers eligible for inclusion if they have had at least 1 hr per week of regular face-to-face contact with the person with dementia. Persons with dementia with less than 1 month of life expectancy are excluded.

### Implementation of the Intervention: Systems Representation

**Figure 2.**
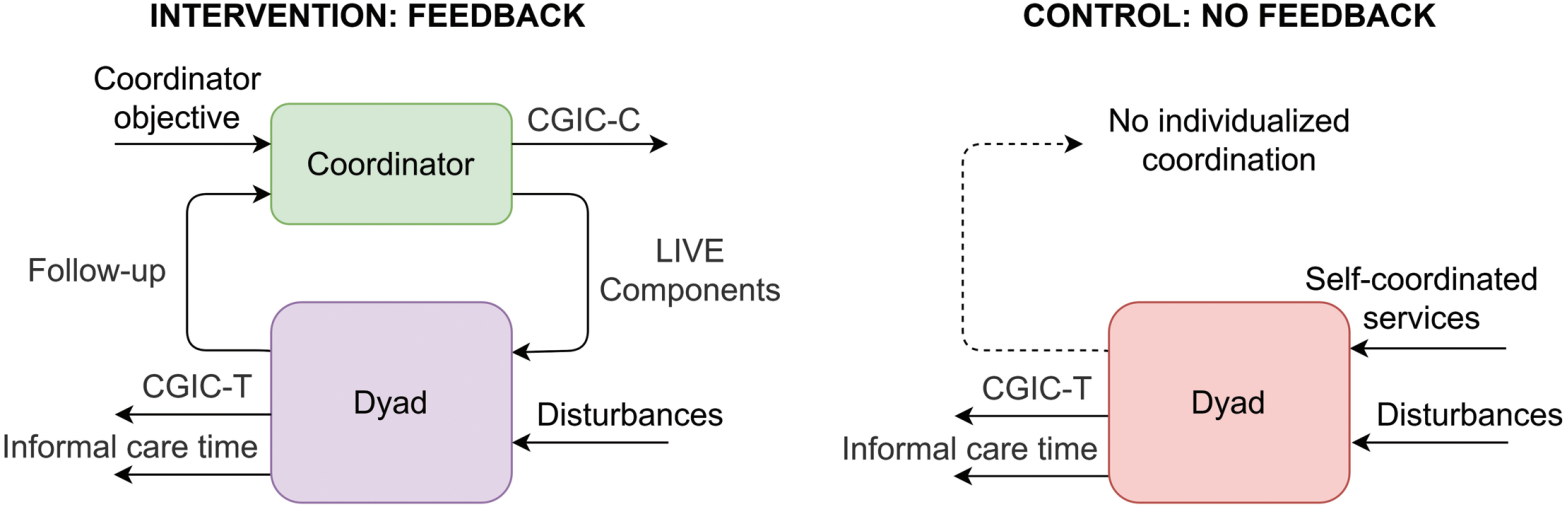
Systems representation of the intervention mechanism. *LIVE *= Learning, Innovation, Volunteering, Empowerment; *CGIC-T *= Clinical Global Impression of Change scale answering the question “On a scale from –5 to +5, how do you perceive the change in self-perceived caregiver situation compared to 6 months ago?” *CGIC-C* answering the question “On a scale from –5 to +5, how do you perceive the contact person in the municipality compared to 6 months ago?”

In systems analysis, a performance indicator is defined as an outcome describing the functioning of the system relative to a preestablished objective, standard, or baseline (Åström & Murray, 2021). For a standard RCT, the intervention mechanism is modeled as two systems: one with feedback for the dyads in intervention period, and one as a single system without feedback for the control period (Figure 2). In this study, the components encompassing the systems are the dyad and the coordinator.

The *coordinator* is a municipality staff member with professional healthcare education (learning disability nurses, occupational therapists, and nurses specialized in dementia care) working in home-based care services or municipality-based memory teams. The LIVE intervention is implemented as a two-stage process: first to the coordinators from the research team composed of medical doctors (geriatric psychiatrist, palliative care physician, and a resident physician), nurses (specialized in nutrition, and primary healthcare systems), and a social scientist specialized in volunteer work among older adults; and second, from coordinators to the dyads. The *Coordinator objective* (Figure 2) models the purpose of the coordinator in the two-system interaction, which is to improve the care situation, for example, reduce burden and psychosocial health, increase quality of life, etc. This purpose is given to the coordinator by the research team through a two-day seminar, training material, and a checklist to record the intervention components offered per dyad. The *performance indicator* of the coordinator is the *Coordinator Clinical Global Impressions of Change* (*CGIC-C*, described in *Measurements* section), including timely follow-up, meeting the needs of dyads, etc. The initialization of the feedback system marks the start of the intervention period when the coordinator visits each dyad at home and introduces them to the specific components of the LIVE intervention. During the intervention, the coordinator acts upon the governed system (dyad) by first selecting the *LIVE Components* most appropriate for the dyad’s specific context, and then by informing the dyad of these concepts (i.e., the coordinator *offers* services to the dyad who is able to exercise their own autonomy in choosing to follow through with the offer or not). The *LIVE Components* are services executed by third-party providers, grouped into:

- Learning: delivers tailored dementia educational programs to persons with dementia (if possible) and informal caregivers by professionals in the field (dementia nurses, geriatricians, and law practitioners), discussing dementia etiology, symptoms and course, strategies to cope with everyday challenges such as BPSD, finances, legal rights, and health and care services.
- Innovation: the coordinators assess the current access to and use of assistive technology, as well as provide information about other relevant technology available in the municipality. In the LIVE@Home.Path trial, assistive technology refers to communication and tracking devices, sensors, and everyday technology such as electronic pill boxes, door locks, and timers on electronic devices (Puaschitz et al., 2021).
- Volunteer support: assesses the attitudes and needs for volunteer involvement in dyads and provides help in applying for support. Volunteers in the trial are members of the local community, who are registered in nonprofit organizations such as The Red Cross and The Norwegian Association for Public Health (The Norwegian Association for Public Health, 2019; The Red Cross, 2019). Volunteering involves spending time with the person with dementia, with the aim of promoting social participation and maintaining meaningful activities.
- Empowerment: includes the systematic medication review and evaluation of values and future wishes (Advance Care Planning) by the general practitioners (GP). In practice, care coordinators inform GPs through the journal system that the person with dementia participates in the trial and motivates them, with help from the caregivers, to contact their GPs for an appointment. Additionally, care coordinators provide the GPs with summary scores from the baseline clinical assessment measurements, for example, BPSD, depression, and caregiver burden.

The feedback is represented by the *Follow-up*, which consists of monthly check-ins by the coordinator, in-person and/or by telephone, and at least two home visits to the dyad during the 6-month intervention period (Husebo et al., 2020). The *Follow-up* can also be triggered by the dyad, who contacts the coordinator as needed.

The *dyad* is formed by the person with dementia and their informal caregiver (Figure 2). They are affected by *Disturbances* (see Author Note), encompassing changes in their lives, for example, disease progression, physical functioning, other circumstances that affect the nominal behavior of a dyad. The performance indicators of the dyad are the *Informal care time* and *Total Clinical Global Impressions of Change* (*CGIC-T*; both described in section *Measurements*). The dyad randomized to the *intervention period* receives the *LIVE Components* from the coordinator in the form of a tailored list of services most suitable to their situation and needs. The list is accompanied by information and practical help for applying for the services. At the same time, the *Follow-up* constitutes the ongoing feedback loop between the coordinator and the dyad during the intervention period. In comparison, participants randomized to the *control period self-coordinate* their own choice of services, which may or may not be optimal for their situation.

### Measurements

#### Primary outcome variables

Informal care time is assessed by the Resource Utilization in Dementia (RUD) instrument, which has shown good validity and reliability in home-dwelling persons with dementia (Wimo & Nordberg, 2007; Wimo et al., 2010). RUD assesses total care hours that informal caregivers have provided in the past 30 days in basic *ADL* (e.g., functional mobility, toileting, hygiene, and eating), instrumental ADL (*IADL*, e.g., medication, preparing meals, household chores) and supervision to prevent adverse events (Wimo et al., 2010). As in previous research, a limit is imposed of 18 hr of informal care per day in *ADL* and *IADL*, to account for 8 hr of sleep (Wübker et al., 2015).

To assess the trial participants’ evaluation of change, a modified CGIC scale is used. Originally, the 9-point scale registers clinically significant change in patients’ health situation after pharmacological treatment in clinical trials (Schneider & Olin, 1996). In the LIVE@Home.Path trial, the scale is adapted to 11 points ranging from –5 = “Much worse” to +5 = “Much better,” with 0 = “No Change” (Husebo et al., 2020). In the present study, we include two variables derived from CGIC: (1) *CGIC-T* answering the question “On a scale from –5 to +5, how do you perceive the change in caregiver situation compared to 6 months ago?” and (2) *CGIC-C* answering the question “On a scale from –5 to +5, how do you perceive the *contact person* in the municipality compared to 6 months ago?” (for the intervention period, this contact person is the coordinator; for the control period, if the contact person exists, they might be, e.g., a registered nurse or an auxiliary nurse working in home-based care).

We also include data from the CGIC scale for each component of the LIVE intervention, with the following question: “On a scale from –5 to +5, how do you perceive the change in Learning/Innovation/Volunteer support/Empowerment compared to 6 months ago?” Change in learning in the context of this trial refers to knowledge about dementia, services, and strategies for coping. Change in innovation refers to making the home environment more suitable through the use of assistive technology. Change in volunteering refers to connecting with new people from volunteer services, providing meaningful activities, and respite to the caregivers. Change in Empowerment refers to communication between the dyad and the GP regarding medication use and advance care planning.

#### Secondary outcomes and covariables

Covariables at baseline include demographic data of the caregivers and persons with dementia (age and gender) and cohabitation status (yes/no). The Physical Self-Maintenance Scale (PSMS), range 6–30, is used for the level of physical self-care (Lawton, 1988). Higher scores indicate reduced ability to self-care (Lawton, 1988). BPSD are assessed by the Neuropsychiatric Inventory (NPI), a 12-item scale, range 0–144, measuring the frequency and intensity of BPSD (delusions, hallucinations, agitation/aggression, dysphoria, anxiety, euphoria, apathy, disinhibition, irritability/lability, and aberrant motor activity, nighttime behavioral disturbances, and appetite and eating abnormalities). Higher NPI scores indicate higher severity and frequency of BPSD (Cummings, 1997). The NPI scale has previously shown good reliability and validity in the Norwegian population (Selbaek et al., 2008).

#### System-related outcomes

**Figure 3.**
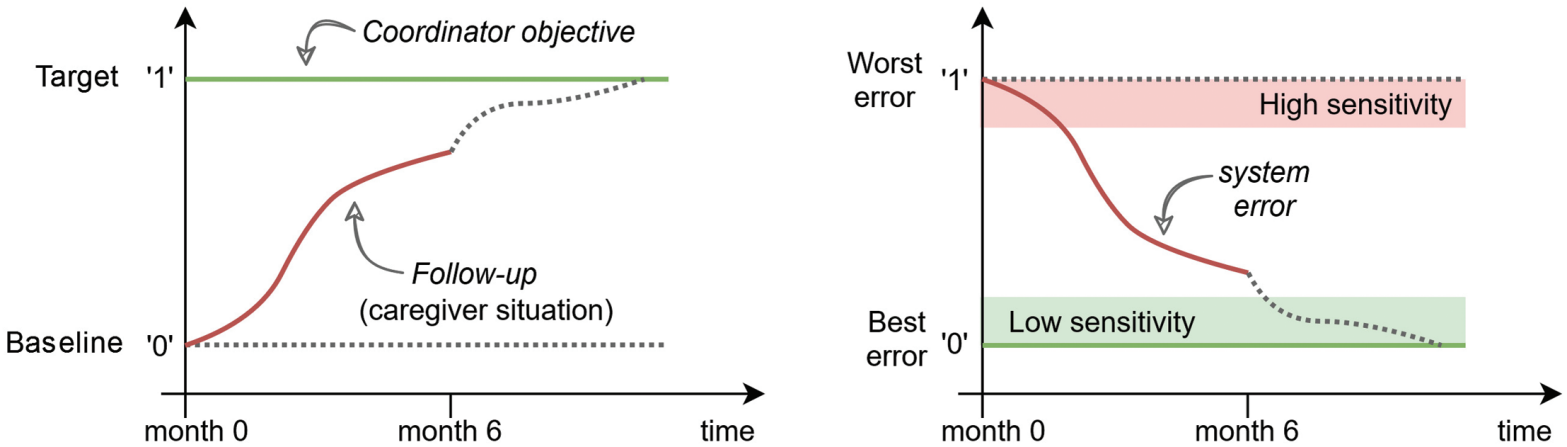
Concept of *system error* relative to the *Coordinator objective* and *Follow-up*; and representation of the high and low sensitivity zones based on *system error* value.

The *system error* is defined as the difference over time between the *Coordinator objective* and the *Follow-up*, which addresses how far from the target the caregiver situation is for a dyad (Figure 3). Supplementary Material, Section 1, provides additional details on the meaning and calculation of the *system error*. Numerically, the *system error* we calculate in this study at a given timepoint is equal to the *sensitivity* of the feedback system at that timepoint (Åström & Murray, 2021), and it is computed from quantifiable outcome measures (the target specified through the *Coordinator objective* vs the value of the outcome measure for the dyad). We choose the primary outcome variables to compute two system errors at the 6-month timepoint: (1) *global impression of change error* (*EG*) based on *CGIC-T* and the number of received *LIVE Components* (total of maximum 5); (2) *care time error* (*ET*) based on change in informal care time domains relative to baseline and number of received *LIVE Components*. The *sensitivity* of the feedback system quantifies the resilience of the system when affected by *Disturbances*: the more sensitive, the less resilient the system is.

## Analysis

System analysis is applied to evaluate the effect of the intervention. The preparatory steps are system representation and definition of performance indicators (both performed in *Method* section). The analysis evaluates the system with feedback (intervention dyad) versus the system without feedback (control dyad) and complies with the RCT design steps: (1) instantiate the intervention and control dyads to account for as much variety in characteristics as possible (randomization and recruitment); (2) observation of functioning (6-month data collection); and (3) groupwide comparison between intervention and control using the chosen performance indicators. To assess the differences between dyads in the intervention and control period, we apply the independent Student’s *t* test, Pearson chi-square test, or the Mann–Whitney *U* test, depending on the structure and distribution of the data. The confidence intervals for the system analysis are defined by the multiple dimensions described by the standard deviations in participant characteristics (Table 1).

**Table 1.** Participant Characteristics Regarding Demographics and Function Levels at the Baseline, by Period (*N* = 225)

Participant characteristics	Total (*N* = 225)	Control (*n* = 165)	Intervention (*n* = 60)	Difference, *p* value
Persons with dementia characteristics				
Age, mean (*SD*)	81.8 (6.9)	81.1 (7.0)	83.5 (6.6)	**.01**
Gender, female, *n* (%)	141 (62.7)	101 (61.2)	40 (66.7)	.47
Living, *n* (%):				
Alone	104 (46.2)	70 (42.4)	34 (56.7)	.11
Spouse	109 (48.4)	84 (50.9)	25 (41.7)	.12
Child	3 (1.3)	1 (1.2)	1 (1.67)	.82
Do you have a volunteer? Yes, *n* (%)	11 (4.9)	9 (5.5)	2 (3.3)	.50
Do you have health technology? Yes, *n* (%)	158 (70.9)	117 (71.8)	41 (68.3)	.61
Have you had a medication review? Yes, *n* (%)	113 (50.7)	83 (50.9)	30 (50.0)	.90
NPI, median [IQR]	12 [4; 24]	12 [3;20]	15 [5; 26]	.19
PSMS, mean (*SD*)	10.1 (3.0)	9.8 (2.8)	10.7 (3.6)	.16
MMSE, mean (*SD*)	20.7 (3.8)	20.6 (3.9)	21.0 (3.6)	.46
Informal care time, hours per day, mean (*SD*)				
ADL	1.5 (1.5)	1.6 (1.6)	1.3 (1.5)	.64
IADL	2.1 (1.9)	2.2 (2.0)	2.0 (1.6)	.69
Number of municipality healthcare—or support services, median [IQR]	1 [1; 2]	1 [0; 2]	1 [1; 2]	.81
Caregiver characteristics				
Age, mean (*SD*)	65.9 (12.2)	65.9 (12.4)	66.0 (11.8)	.98
Living with the caregiver, yes, *n* (%)	105 (46.7)	81 (49.1)	24 (40.0)	.23
Gender, female *n* (%)	149 (66.2)	107 (64.9)	42 (70.0)	.47
Relationship, *n* (%)				.55
Spouse/partner	98 (43.6)	75 (45.5)	23 (38.3)	
Child	114 (50.7)	80 (48.5)	34 (56.7)	
Other	13 (5.8)	10 (6.1)	3 (5.0)	
Education, *n* (%)				.36
Primary school	13 (5.8)	9 (5.5)	4 (6.7)	
Secondary/vocational school	59 (26.2)	47 (28.5)	12 (20.0)	
Higher education	147 (65.3)	103 (62.4)	44 (73.3)	
Working, yes, *n* (%)	112 (49.8)	81 (49.1)	31 (51.7)	.89
Have had a dementia education course before? Yes, *n* (%)	43 (19.6)	34 (21.3)	9 (15.0)	.29

*Notes*: Bold value: significant *p* value. ADL = Activities of daily living (e.g., toileting, personal hygiene, and meal situations); IADL = Instrumental activities of daily living (e.g., taking medicine and out-patient visits); IQR = interquartile range; MMSE = Mini-Mental Status Examination [range 0–30], a lower score indicates greater cognitive impairment; *N* = total sample; *n* = number of patients; NPI = Neuropsychiatric Inventory sum of 12 items; PSMS = Physical Self-Maintenance Scale [range 0–30], higher score indicates lower functional capacity; *SD* = standard deviation. Difference between groups was tested with unequal variances *t* test for normal and Wilcoxon–Mann–Whitney test for nonnormally distributed continuous variables, Pearson chi-square tests for categorical variables.

### Informal Care Time

- *Regression analysis.* We analyze the LIVE intervention effect on change from baseline to 6-month assessment in RUD informal care time (dependent variable) as a binary variable (increase/no change = 0; decrease = 1) in *ADL* and *IADL* separately by using logistic regression analysis. The Akaike Information Criterion and Hosmer–Lemeshow goodness of fit analysis are used for variable and model selection.

### CGIC-T and CGIC-C

- *Total and coordinator intervention effect analysis.* Independent two-sample *t* tests are employed to detect the difference in the *CGIC-T* and *CGIC-C* scores, respectively. Means of the *CGIC-T* and *CGIC-C* scores are used to create the heatmap for visualization.
- *Component-wise intervention effect analysis.* To test the reach of the intervention components, we categorize the study participants into: I—Have never received the intervention component but might have been offered it; II—Have received the component during the intervention period (Note: for Learning, category II also includes receiving the component prior to the intervention start). We then create a heatmap, visualizing the reach of each intervention component and corresponding *CGIC-T* scores for the two categories I and II.

### Whole-System Analysis

- *Error analysis.* The system errors (at month 6) are computed using the equation of the sensitivity function for feedback systems and the Final Value Theorem (Åström & Murray, 2021) as follows: $EG=\;{\left(1+CGIC\text{-}T\cdot LIVE\right)}^{-1}$ and $ET_{i}=\;{\left(1+time_{i}\cdot LIVE\right)}^{-1}$, where *LIVE* is the total number of *LIVE Components* received, and *time*_*i*_ is the change in informal care time calculated per RUD domain: i∈{ADL,IADL}. All variables in the equations are mapped to [0–1] for computational consistency (Note: *time*_*i*_ requires an inversion due to increase in time as a negative outcome, and decrease as positive, thus *time*_*i*_=‘0’ means increase in care time, and ‘1’ means decrease). Differences between the control and intervention in *EG* and *ET*_*i*_ are analyzed using the Mann–Whitney *U* test or *t* test with unequal variances, depending on the data distribution.
- *Sensitivity analysis* considers sensitivity *S*=‘1’ as the baseline of the dyad and *S*=‘0’ as the desired target in the *Coordinator objective* (Figure 3). For the systems analyzed here, the value of the sensitivity relative to the *CGIC-T* score at month 6 is equal to the computed error *EG* at month 6; based on the closeness of the *EG* values to either ‘0’ or ‘1’, the sensitivity of the system to *Disturbances* is assessed for the control and intervention period, by their distribution.
- *Case studies*. When performing the overall analytical sensitivity analysis of the feedback system, the sensitivity function *S* would be calculated mathematically from the dynamic model equations of the comprising systems. In this study, these are the coordinator and the dyad, for which we do not have the necessary differential-equation models because the behavior of human beings is much more complex than can be described through a single-input-single-output model. Moreover, the available sensitivity measurements are for two timepoints (baseline and month 6), and so the function *S* cannot be approximated from data in its entirety. Because sensitivity analysis estimates the resilience of a system to *Disturbances*, which in this study are highly individualized for each participant dyad (e.g., health status of the coordinator, personalities, other familial or workplace events), feedback systems analysis methodology dictates that instances of the system (so-called “use case scenarios”) are selected for contextualized investigation. Therefore, four dyads D1–D4 are chosen from the high and low sensitivity zone presented in Figure 3 (two from the control group and two from the intervention group) and are individually analyzed.

All statistical analyses are performed using Stata IC version 17 and visualization using Matlab 16. Results are considered statistically significant for *p* < .05. Missing values are addressed using listwise deletion.

## Ethics Statement

The LIVE@Home.Path trial is in line with the Declaration of Helsinki: Ethical Principles for Medical Research Involving Human Subjects (World Medical Association, 2001). Informed consent is obtained in direct conversation with the person with dementia (if possible) and his/her relatives. Where capacity to consent in person with dementia is questionable, consent is obtained from the primary caregiver. Due to the General Data Protection Regulation, Article 35 on the Data Protection Impact Assessment (Wolford, 2019) is applied, highlighting the data minimization. The trial is approved by the Regional Committee of Medical and Health Research Ethics (2019/385/REK), the Norwegian Center for Research Data (UiB archive reference 2019/5569), and registered at clinicaltrials.gov (NCT04043364).

## Results

**Figure 4.**
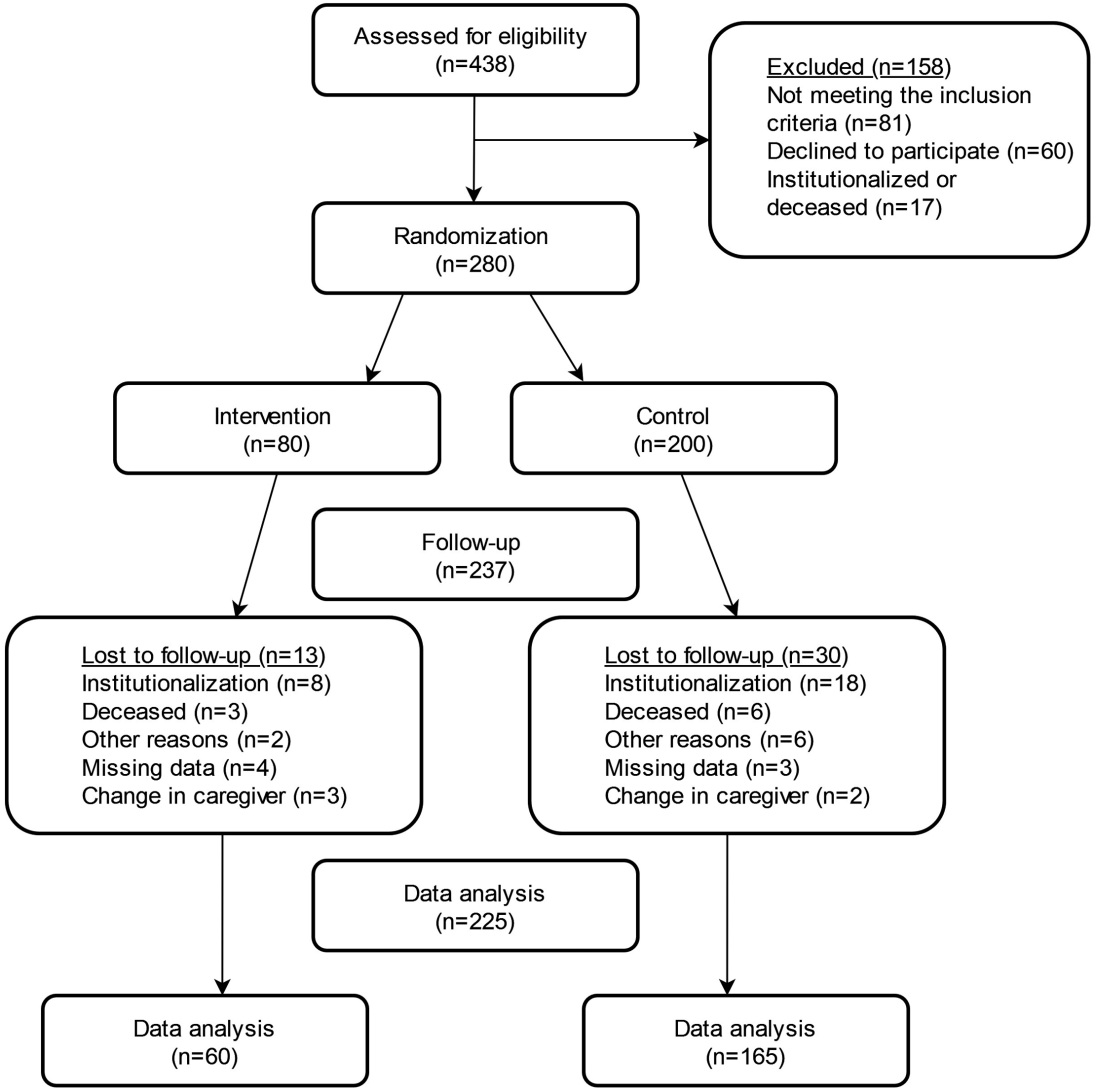
Flow chart of study participants in the LIVE@Home.Path trial.

In total, 438 dyads (*n* = 876) are screened for participation, and 280 (*n* = 560) are included in study participation (Figure 4). At baseline, 200 dyads are randomized to periods 2 and 3 (control), whereas 80 dyads are randomized to period 1 (intervention). Dyads lost to the 6-month follow-up (*n* = 43), dyads with missing >20% of data at baseline or at the 6-month timepoint (*n* = 7), and dyads with changes in the caregiver (*n* = 5) are excluded. Attrition analysis for the dropout is presented in Supplementary Material, Section 2. The final sample in this study includes 225 dyads. The baseline characteristics for the intervention (*n* = 165) and control period (*n* = 60) are presented in Table 1. Mean age of persons with dementia is 81.8 years, 62.7% are female, and mean MMSE score is 20.7. Informal caregivers for the total sample have a mean age of 65.9 years, 66.2% are female, 50.7% are adult children of the persons with dementia, and 46.7% of the caregivers co-reside with the persons with dementia. The co-residing and non-co-residing persons with dementia have no significant clinical differences in terms of physical function, cognitive impairment, and BPSD (data not shown). The mean age of the persons with dementia is slightly higher in the intervention period compared to the control period.

The regression analyses (Table 2) show no statistically significant intervention effect on informal care time reduction. However, we find that, holding all other predictor variables constant, decrease in *IADL* time during the 6-month period is associated with higher caregiver age (OR 1.10, CI [1.00; 1.22], *p* = .04), whereas care time increase is associated with female gender (OR 0.20, CI [0.4; 0.85], *p* = .03) and spousal relationship (OR 0.03, CI [0.00; 0.52], *p* = .02). Decrease in ADL care time is associated with higher NPI scores (OR 1.11, CI [0.87; 1.43], *p* = .02).

**Table 2.** Logistic Regression Analysis of the LIVE Intervention Effect on Decrease in Informal Care Time From Baseline to 6 Months

Decrease = 1 Increase/no change = 0	Model 2 ADL Adjusted OR (95% CI)	*p* Value	Model 3 IADL Adjusted OR (95% CI)	*p* Value
*N*	**83**		**83**	
Treatment (control = 0)	0.41 (0.07; 2.25)	.31	0.72 (0.23; 2.19)	.56
Spouse (other = 0)	0.58 (0.01; 21.09)	.76	0.03 (0.00; 0.52)	**.02**
Caregiver age	0.99 (0.87; 1.13)	.95	1.10 (1.00; 1.22)	**.04**
Caregiver gender (male = 0) Female	0.80 (0.13; 4.92)	.81	0.68 (0.20; 2.3)	.54
Person with dementia age	0.95 (0.81; 1.09)	.46	0.96 (0.86; 1.08)	.55
Person with dementia gender (male = 0) Female	1.47 (0.22; 9.53)	.68	0.20 (0.04; 0.85)	**.03**
MMSE	0.95 (0.76; 1.19)	.67	0.98 (0.85; 1.13)	.82
NPI	1.05 (1.00; 1.10)	**.02**	1.00 (0.97; 1.04)	.65
PSMS	1.11 (0.87; 1.43)	.37	0.95 (0.78; 1.16)	.65
Hosmer–Lemeshow goodness of fit	0.55		0.26	

*Notes*: Bold values: significant *p* value. ADL = Activities of daily living (e.g., taking medicine and out-patient visits); CI = confidence interval; IADL = Instrumental activities of daily living (e.g., medication, preparing meals, and household chores); MMSE = Mini-Mental Status Examination [range 0–30], higher scores indicate better cognitive functioning; NPI 12 = Neuropsychiatric Inventory sum of 12 items; OR = odds ratio; PSMS = Physical Self-Maintenance Scale [range 0–30], higher score indicates lower functional capacity.

Table 3 presents results from the two-sample *t* tests with unequal variances, showing that after the 6-month intervention, *CGIC-T* is significantly higher in the intervention period compared to the control period (*p* < .01). Likewise, *CGIC-C* (Table 3) is significantly higher for the intervention (*p* < .01). Table 3 also shows how caregivers evaluate each of the intervention components: the intervention group reports larger improvements for L (*p* < .01), I (*p* < .01), and E (*p* = .02). There is no significant difference for the V component (p = .07).

**Table 3.** Mean CGIC Scores by Intervention (60) and Control Period (164), Total *N* = 224

Measurements	Study period	Mean, (*SD*)	*t* Value	*p* Value
CGIC-T (dyad performance indicator)	Intervention	0.63 (2.4)	−3.1	<.01
	Control	−0.43 (1.7)		
CGIC-C (coordinator performance indicator)	Intervention	2.2 (2.0)	–10.2	<.01
	Control	0.17 (0.9)		
CGIC-L	Intervention	1.75 (1.9)	−2.8	<.01
	Control	0.97 (1.8)		
CGIC-I	Intervention	1.1 (1.6)	−2.8	<.01
	Control	0.45 (1.2)		
CGIC-V	Intervention	0.58 (1.2)	−1.8	.07
	Control	0.26 (0.9)		
CGIC-E	Intervention	0.93 (1.6)	−2.3	.02
	Control	0.39 (1.3)		

*Notes*: Unequal variances *t* test was used to test differences in groups. CGIC-C = Coordinator Clinical Global Impressions of Change; CGIC-T = Total Clinical Global Impression of Change; CGIC-L = Learning Clinical Global Impressions of Change; CGIC-I = Assistive technology Clinical Global Impressions of Change; CGIC-V = Volunteering Clinical Global Impressions of Change; CGIC-E = Empowerment Clinical Global Impression of Change; *n* = number of caregivers; *N* = total sample; *SD* = standard deviation.

**Figure 5.**
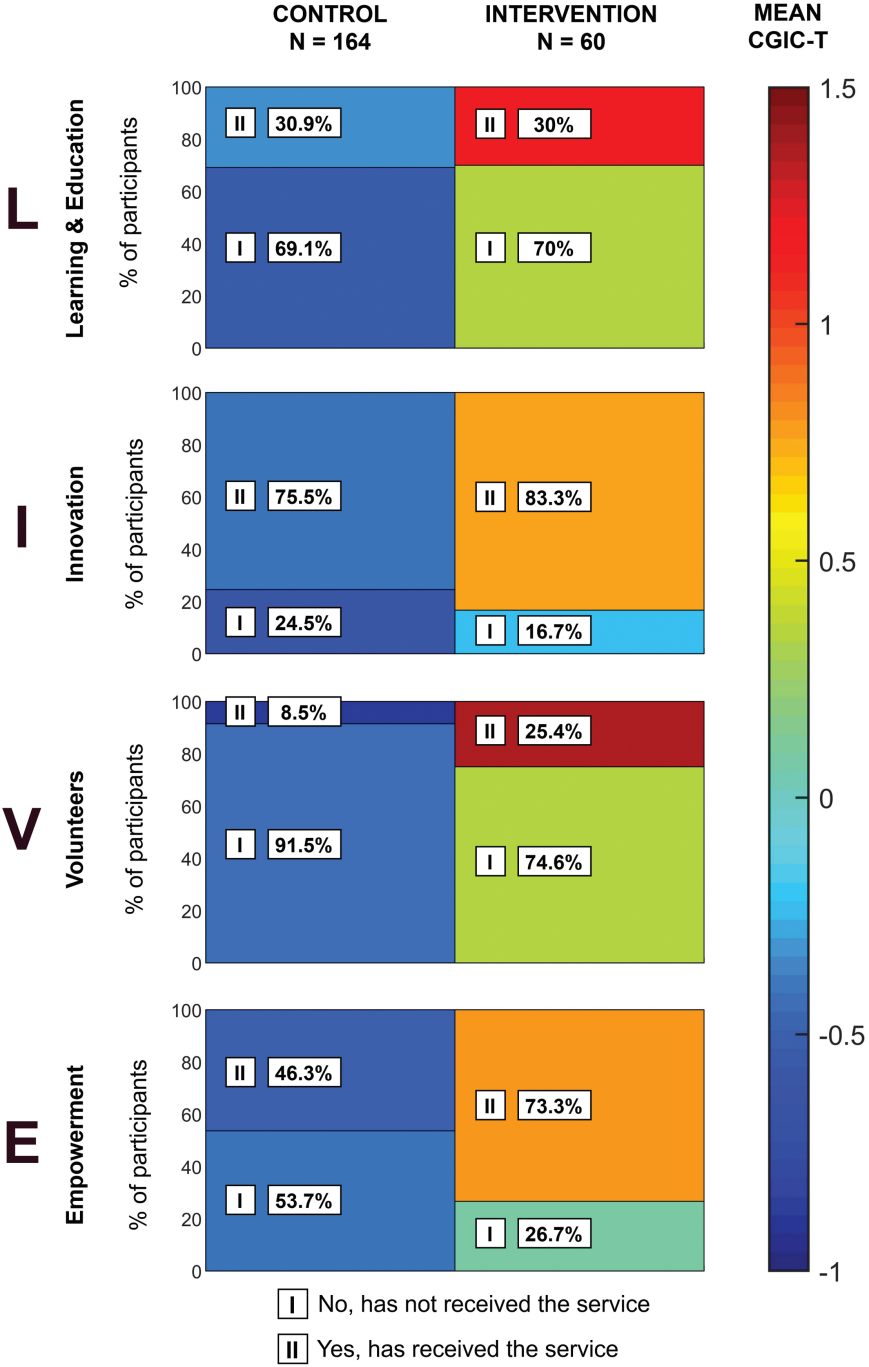
Heatmap demonstrating the received intervention components and the mean CGIC-T score by control (164) and intervention period (60), *N* = 224. *CGIC-T =* Clinical Global Impression of Change presenting the question: “On a scale from –5 to +5, how do you perceive the change in self-perceived caregiver situation compared to 6 months ago?”; Empowerment combines two *LIVE Components*: Medication review and Advance Care Planning.

Figure 5 presents the reach of the intervention components as a heatmap. It shows that the proportion of the dyads who received the *LIVE Components* is significantly higher in the intervention period for volunteering (*p* < .01) and empowerment (*p* < .01), while as presented in Table 1, there are no differences at the baseline. The dyads in the intervention period consistently score higher on CGIC-T compared to the controls, even if they did not receive all of the components.

The *EG* error analysis for the whole system shows significant differences between the intervention (0.56, *SD* 0.03) and control means (0.74, *SD* 0.01, *p* < .01). We also find the following differences in *ET*_*i*_: *ADL* intervention 0.48 (*SD* 0.26) versus control 0.61 (*SD* 0.20, *p* < .05), and *IADL* intervention 0.52 (*SD* 0.25) versus control 0.68 (*SD* 0.18, *p* < .01). When separately analyzing the scaled *time*_*i*_ variables (per RUD domain: i∈{ADL,IADL}) between intervention and control, we find no significant difference (data not shown), verifying the findings from the regression analysis.

The sensitivity analysis shows that the skewness of the control period distribution is −0.67, indicating that the control period has moderately right-skewed data, clustering most dyads near the high sensitivity zone. The skewness of the intervention period is −0.08, indicating that the distribution is approximately symmetric. Four dyads are selected for single observations case studies from the ends of the sensitivity interval: D1 and D3 have low sensitivity (closest to ‘0’), whereas D2 and D4 have high sensitivity (equal to ‘1’; Figure 3). Table 4 contains the characteristics of the dyads.

**Table 4.** Characteristics of the Dyads Included in the Case Studies, *N* = 4

Dyad	D1	D2	D3	D4
Type of dementia	Alzheimer	Unspecified	Alzheimer	Alzheimer
Person with dementia gender	Male	Female	Male	Male
Symptom onset (years)	6	6	4	2
Caregiver gender	Female	Male	Female	Female
Caregiver level of education	Higher education	Higher education	Higher education	Primary and lower secondary
Living together	Yes	Yes	Yes	Yes
Treatment	Intervention	Intervention	Control	Control
*EG* ^a^	0.052	1	0.219	1
*E*_time_ ^b^				
* ADL*	0.24	1	0.30	1
* IADL*	Missing	1	0.31	1
MMSE total	15	25	18	Missing
Difference between baseline and 6 month follow-up score				
NPI	21	0	−2	0
PSMS	5	Missing	0	1
CGIC-C	4	5	4	0
CGIC-T	4	0	3	−1
Number of LIVE components^c^	5	0	4	0

*Notes*: ADL = Activities of daily living (e.g., personal hygiene, bathing, and dressing); CGIC-C = Coordinator Clinical Global Impressions of Change; CGIC-T = Total Clinical Global Impression of Change; IADL = Instrumental activities of daily living (e.g., taking medicine and out-patient visits); MMSE = Mini-Mental Status Examination; NPI = Neuropsychiatric Inventory sum of 12 items; PSMS = Physical Self-Maintenance Scale.

^a^Global impression of change error.

^b^Informal care time change error.

^c^Number of LIVE components received.

### Case Studies

#### Case study 1

D1 is a dyad formed of a married couple, with the first symptoms of Alzheimer’s disease appearing 6 years before. The dyad belongs to the intervention group and comes closest to the *Coordinator objective* (*EG* = 0.05) in the overall sample. Although BPSD increases and physical function decreases, they report an improvement in caregiver situation (*CGIC-T*). The dyad receives all the *LIVE Components*, three of them during the 6-month period, and reports a positive perception of the coordinator performance (*CGIC-C*).

#### Case study 2

D2 is a dyad with 6 years of symptoms. D2 scores high on the coordinator performance, even though they have not opted for any of the components during the intervention period. Note that the caregiver has higher education in healthcare (not specified in the table) and appreciates the communication with the coordinator. However, it might mean that the offered *LIVE Components* were not suitable for this particular dyad and this case can be an excellent example of “one size does not fit all.” Moreover, it also shows that the *Coordinator objective* should be individually tailored and not generalized for entire groups.

#### Case study 3

D3 is a dyad with a caregiver with higher education, and symptoms that began 4 years before enrollment in the trial. They have received four of the five *LIVE Components*, three during the last six months, although they have been in the control group. This might be a good example where the “primary contact person” follow-up at the municipality functions well because the dyad comes very close to the *Objective* assigned to the coordinator in the intervention group. The dyad experiences an overall improvement in BPSD and reports a positive impression of change.

#### Case study 4

D4 is a dyad with a short illness duration in the control group. They report no follow-up from the home-based services or coordinator. Although there is only a small decrease in physical functioning, the self-perceived care situation for the caregiver worsens over 6 months. This specific case is a good example of the need for systematic and timely follow-up, which is beneficial to both the persons with dementia and their caregivers.

## Discussion

This study shows that compared to the control period, informal caregivers randomized to the LIVE intervention period report higher satisfaction rates with their care situation for the home-dwelling person with dementia. These caregivers value the ongoing support and the coordinator’s performance positively. Moreover, they report a significantly higher improvement rate in their knowledge of dementia, healthcare technology, and communication with GPs for persons with dementia. Caregiver time does not decrease during the intervention period. Our findings are of key importance for municipality healthcare personnel, stakeholders, and politicians, as they highlight the value of a coordinated care process and tailored healthcare policy for persons with dementia, which also benefits their informal caregivers.

In line with our hypothesis, we find that the LIVE intervention improves the caregiver situation evaluated by *CGIG-T*. We consider all intervention components through their separate scores on the *CGIC-T* scale. The heatmap in Figure 5 shows that the reach of the components is higher in the intervention period and that the perception of the caregiver’s own care situation is better for dyads who have received *LIVE Components.* Interestingly, it also shows that the intervention dyads who choose not to pursue any of the *LIVE Components* are still reporting better perceived caregiver situation than their control counterparts. This is due to the presence of the safety net secured by the coordinator, as also reflected by increased *CGIG-C* during the intervention. A cross-national synthesis including eight European countries (*n* = 261) exploring facilitators and barriers of formal care use among persons with dementia reveals that dementia coordinators are an essential aspect of care continuity, facilitate the use of formal care, prevent uncertainty, and provide a higher sense of security (Stephan et al., 2018). These results comply with the findings of perceived safety net created by the coordinator described by Fæø et al. (2020). Systematic reviews demonstrate that case management/care coordinator interventions have a positive effect on caregiver psychological health and well-being, unmet needs, burden, and anxiety and improve dementia knowledge (Backhouse et al., 2017; Cheng et al., 2020; Walter & Pinquart, 2020; You et al., 2012). However, some studies also report the opposite, which might be attributed to, for example, small sample sizes, timeliness of the coordinator, etc. (Jansen et al., 2011; Tanner et al., 2015), and barriers to sufficient intervention implementation (Khanassov et al., 2014).

However, contrary to our second hypothesis, our findings do not demonstrate any significant decrease in time spent on caregiving tasks during the intervention period. This might be explained by the increased caregiver awareness as they realized their total engagement in different caregiver tasks. Although the caregiver time does not decrease during the intervention, the system error analysis shows that the intervention group comes closer to the desired *Coordinator objective* compared to care as usual. This indicates that even though the number of care hours are fairly equal or increase during the course of the disease, the caregivers may not perceive it as increasingly burdensome due to the safety net created by the care coordinator. This is supported by Lindt et al. (2020), who state that perceived caregiver situation can be alleviated by social support, either from other family caregivers or healthcare professionals. Our results show that the interactive two-way communication and feedback between the dyad and the care coordinator significantly improves the caregiver situation, which may potentially contribute to better mental and physical well-being in informal caregivers.

Because care pathways are complex interventions in complex systems (Seys et al., 2019), we apply a feedback system analysis approach for evaluating the intervention effect on perceived caregiver situation and care time. The regression analysis, looking at the unidirectional relationship between treatment and change in informal care time, shows no significant intervention effect. However, the system error analysis (*ET*_*i*_) accounts for the overall perspective and for the number of intervention components received in total. The system error demonstrates a difference between the control and intervention groups and that the intervention group comes closer to the desired *Coordinator objective* compared to care as usual. The sensitivity value (*S*) quantifies the resilience of the feedback system to *Disturbances*, ranging from 0 to 1: the closer to zero, the better the resilience. Results show that approximately 50% of the dyads in the intervention group score between *S* = 0.05 and *S* = 0.58, whereas in the control group, only 10% of the dyads score between *S* = 0.28 and *S* = 0.51. For this study, better resilience means that the dyads are more likely to adapt to smaller changes in their daily lives, for instance, small variations in BPSD. This situation is illustrated by dyad D1, which experiences an increase in BPSD, but still reports improvement in caregiver situation. In contrast, dyad D4 in control group shows worsening of the caregiver situation and reports no follow-up from the municipality, which makes them less resilient (*S* = 1). By calculating more sensitivity values in future research, we could describe or predict the likelihood of permanent nursing home admission. Although we cannot test this hypothesis in this study, it is a worthy avenue to explore. Both the system error and the sensitivity suggest that the feedback system is an appropriate analysis approach to evaluate RCTs with supervised adaptive interventions.

### Strengths and Limitations

The main strength of the study is that the effect of the intervention components considers changes in real-world settings, allowing the intervention to be tailored for individual needs of a dyad as a unit. Moreover, healthcare professionals, caregivers, and user-representatives are involved in the development and implementation of the LIVE@Home.Path trial to enhance the feasibility and acceptability in clinical practice (Fæø et al., 2020). The trial design stipulates that the coordinator provides *Follow-up* with a time-step smaller than the data collection time-step (6 months), making it suitable for system analysis because the collected outcome measures consider the entire evolution of the system over those 6 months.

This study has some methodological limitations. Although this is a multisite trial with a relatively large sample size, yielding high generalizability of the results, the imbalanced sample size compromises the statistical power of the results in the current study. The intervention to control group ratio of 1:2 leads to modest reduction of power in statistical analysis; however, this does not affect the system analysis approach. Assessing informal care time with the RUD scale is prone to inaccuracy due to the recall method, as direct observations and caregiver diaries are considered as the gold standard of time recording (Van den Berg & Spauwen, 2006). Nevertheless, the RUD assessment tool has shown accurate estimation of the time provision and is considered a valid and reliable substitution for direct observations (Wimo & Nordberg, 2007; Wimo et al., 2013). We might underestimate the amount of informal care time in some care domains due to missing data. The trial uses convenience sampling, and some dyads utilized formal care services and received the intervention components (e.g., learning) before the intervention started. This might leave little room for improvements and might result in a reduced intervention effect. Moreover, Norwegian municipality-based dementia care teams and the municipality administrative unit responsible for healthcare service distribution are a low-threshold service in Norway, which dyads in the control group have easy access to. However, LIVE@Home.Path makes a distinction that the active intervention differs from usual care, as it is delivered by a designated coordinator applied in the context of the trial. The trial strives to minimize the intervention contamination by introducing the trial design to the participants at baseline (Magill et al., 2019). We can only calculate the system sensitivity for one timepoint (month 6), which does not fully incorporate the whole predictive power of the sensitivity function. Further research is needed to investigate the sensitivity of the system for other data points over a longer period of time.

## Conclusion

In conclusion, this study demonstrates the impact of the multicomponent LIVE intervention on the self-perceived caregiver situation and supports the policy of a key person to provide support. We show that feedback system analysis is a promising method to investigate complex intervention trials, such as LIVE@Home.Path. Meeting the needs of persons with dementia and their caregivers is a complex process that requires coordinated input from health and social care services, the voluntary sector, government policies, and user communities.

## Supplementary Material

igae020_suppl_Supplementary_Material

## Data Availability

The data sets used and/or analyzed during the current study are available from the corresponding author upon reasonable request.

## Author Note

In system analysis terminology, disturbances define all factors that affect the underlying system functioning. Åström, K. J., & Murray, R. M. (2021). *Feedback systems: An introduction for scientists and engineers*. Princeton university press.

## References

[CIT0001] American Association of Retired Persons. (2021). *2021 Home and community preferences survey: A national survey of adults age 18‐Plus*. https://www.aarp.org/research/topics/community/info-2021/2021-home-community-preferences.html

[CIT0002] Amjad, H., Wong, S. K., Roth, D. L., Huang, J., Willink, A., Black, B. S., Johnston, D., Rabins, P. V., Gitlin, L. N., Lyketsos, C. G., & Samus, Q. M. (2018). Health services utilization in older adults with dementia receiving care coordination: The MIND at home trial. Health Services Research, 53(1), 556–579. 10.1111/1475-6773.12647 28083879 PMC5785326

[CIT0003] Angeles, R. C., Berge, L. I., Gedde, M. H., Kjerstad, E., Vislapuu, M., Puaschitz, N. G., & Husebo, B. S. (2021). Which factors increase informal care hours and societal costs among caregivers of people with dementia? A systematic review of Resource Utilization in Dementia (RUD). Health Economics Review, 11(1), 37. 10.1186/s13561-021-00333-z 34536149 PMC8449888

[CIT0004] Åström, K. J., & Murray, R. M. (2021). Feedback systems: An introduction for scientists and engineers. Princeton University Press.

[CIT0005] Backhouse, A., Ukoumunne, O. C., Richards, D. A., McCabe, R., Watkins, R., & Dickens, C. (2017). The effectiveness of community-based coordinating interventions in dementia care: A meta-analysis and subgroup analysis of intervention components. BMC Health Services Research, 17(1), 717. 10.1186/s12913-017-2677-2 29132353 PMC5683245

[CIT0006] Brodaty, H., & Donkin, M. (2009). Family caregivers of people with dementia. Dialogues in Clinical Neuroscience, 11(2), 217–228. 10.31887/DCNS.2009.11.2/hbrodaty 19585957 PMC3181916

[CIT0008] Burton, C., Elliott, A., Cochran, A., & Love, T. (2018). Do healthcare services behave as complex systems? Analysis of patterns of attendance and implications for service delivery. BMC Medicine, 16(1), 138. 10.1186/s12916-018-1132-5 30189866 PMC6127924

[CIT0009] Cheng, S. -T., Li, K. -K., Losada, A., Zhang, F., Au, A., Thompson, L. W., & Gallagher-Thompson, D. (2020). The effectiveness of nonpharmacological interventions for informal dementia caregivers: An updated systematic review and meta-analysis. Psychology and Aging, 35(1), 55–77. 10.1037/pag0000401 31985249

[CIT0010] Chow, S., Chow, R., Wan, A., Lam, H. R., Taylor, K., Bonin, K., Rowbottom, L., Lam, H., DeAngelis, C., & Herrmann, N. (2018). National dementia strategies: What should Canada learn? Canadian Geriatrics Journal, 21(2), 173–209. 10.5770/cgj.21.299 29977433 PMC6028171

[CIT0011] Clark, A. M. (2013). What are the components of complex interventions in healthcare? Theorizing approaches to parts, powers and the whole intervention. Social Science & Medicine, 93(185), 185–193. 10.1016/j.socscimed.2012.03.035 22580076

[CIT0012] Cummings, J. L. (1997). The Neuropsychiatric Inventory: Assessing psychopathology in dementia patients. Neurology, 48(5 Suppl 6), 10S–16S. 10.1212/wnl.48.5_suppl_6.10s 9153155

[CIT0013] Ekman, B., McKee, K., Vicente, J., Magnusson, L., & Hanson, E. (2021). Cost analysis of informal care: Estimates from a national cross-sectional survey in Sweden. BMC Health Services Research, 21(1), 1236. 10.1186/s12913-021-07264-9 34781938 PMC8591811

[CIT0014] Fæø, S. E., Tranvåg, O., Samdal, R., Husebo, B. S., & Bruvik, F. K. (2020). The compound role of a coordinator for home-dwelling persons with dementia and their informal caregivers: Qualitative study. BMC Health Services Research, 20(1), 1045. 10.1186/s12913-020-05913-z 33198779 PMC7670600

[CIT0015] Gedde, M. H., Husebo, B. S., Vahia, I. V., Mannseth, J., Vislapuu, M., Naik, M., & Berge, L. I. (2022). Impact of COVID-19 restrictions on behavioural and psychological symptoms in home-dwelling people with dementia: A prospective cohort study (PAN. DEM). BMJ Open, 12(1), e050628. 10.1136/bmjopen-2021-050628 PMC8787843 35074810

[CIT0016] Gillespie, R., Mullan, J., & Harrison, L. (2014). Managing medications: The role of informal caregivers of older adults and people living with dementia. A review of the literature. Journal of Clinical Nursing, 23(23-24), 3296–3308. 10.1111/jocn.12519 24354583

[CIT0017] Hemming, K., Haines, T. P., Chilton, P. J., Girling, A. J., & Lilford, R. J. (2015). The stepped wedge cluster randomised trial: Rationale, design, analysis, and reporting. British Medical Journal, 350, h391. 10.1136/bmj.h391 25662947

[CIT0018] Husebo, B. S., Allore, H., Achterberg, W., Angeles, R. C., Ballard, C., Bruvik, F. K., Fæø, S. E., Gedde, M. H., Hillestad, E., Jacobsen, F. F., Kirkevold, O., Kjerstad, E., Kjome, R., Mannseth, J., Naik, M., Nouchi, R., Puaschitz, N., Samdal, R., Tranvåg, O., & Berge, L. I. (2020). LIVE@ Home. Path—innovating the clinical pathway for home-dwelling people with dementia and their caregivers: Study protocol for a mixed-method, stepped-wedge, randomized controlled trial. Trials, 21(1), 510. 10.1186/s13063-020-04414-y 32517727 PMC7281688

[CIT0019] Jansen, A. P. D., van Hout, H. P. J., Nijpels, G., Rijmen, F., Dröes, R. -M., Pot, A. -M., Schellevis, F. G., Stalman, W. A. B., & van Marwijk, H. W. J. (2011). Effectiveness of case management among older adults with early symptoms of dementia and their primary informal caregivers: A randomized clinical trial. International Journal of Nursing Studies, 48(8), 933–943. https://doi.org /10.1016/j.ijnurstu.2011.02.004 21356537 10.1016/j.ijnurstu.2011.02.004

[CIT0020] Khanassov, V., Vedel, I., & Pluye, P. (2014). Barriers to implementation of case management for patients with dementia: A systematic mixed studies review. Annals of Family Medicine, 12(5), 456–465. 10.1370/afm.1677 25354410 PMC4157983

[CIT0021] König, H. -H., Leicht, H., Brettschneider, C., Bachmann, C., Bickel, H., Fuchs, A., Jessen, F., Köhler, M., Luppa, M., Mösch, E., Pentzek, M., Werle, J., Weyerer, S., Wiese, B., Scherer, M., Maier, W., & Riedel-Heller, S. G.; AgeCoDe Study Group (2014). The costs of dementia from the societal perspective: Is care provided in the community really cheaper than nursing home care? Journal of the American Medical Directors Association, 15(2), 117–126. 10.1016/j.jamda.2013.10.003. 24321877

[CIT0062] Lawton, M. (1988). Physical Self-Maintenance Scale (PSMS) original observer-rated version. Psychopharmacology Bulletin, 24(4), 793–794. 3249787

[CIT0022] Lin, C. Y., Shih, P. Y., & Ku, L. E. (2019). Activities of daily living function and neuropsychiatric symptoms of people with dementia and caregiver burden: The mediating role of caregiving hours. Archives of Gerontology and Geriatrics, 81, 25–30. 10.1016/j.archger.2018.11.009 30496871

[CIT0023] Lindeza, P., Rodrigues, M., Costa, J., Guerreiro, M., & Rosa, M. M. (2020). Impact of dementia on informal care: A systematic review of family caregivers’ perceptions. BMJ Supportive Palliative Care, 1–12. 10.1136/bmjspcare-2020-002242 33055092

[CIT0024] Lindt, N., van Berkel, J., & Mulder, B. C. (2020). Determinants of overburdening among informal carers: A systematic review. BMC Geriatrics, 20(1), 304. 10.1186/s12877-020-01708-3 32847493 PMC7448315

[CIT0025] Lord, K., Livingston, G., Robertson, S., & Cooper, C. (2016). How people with dementia and their families decide about moving to a care home and support their needs: Development of a decision aid, a qualitative study. BMC Geriatrics, 16(1), 68. 10.1186/s12877-016-0242-1 27001704 PMC4802590

[CIT0026] Magill, N., Knight, R., McCrone, P., Ismail, K., & Landau, S. (2019). A scoping review of the problems and solutions associated with contamination in trials of complex interventions in mental health. BMC Medical Research Methodology, 19(1), 4. 10.1186/s12874-018-0646-z 30616508 PMC6323722

[CIT0027] McGill, E., Er, V., Penney, T., Egan, M., White, M., Meier, P., Whitehead, M., Lock, K., Anderson de Cuevas, R., Smith, R., Savona, N., Rutter, H., Marks, D., de Vocht, F., Cummins, S., Popay, J., & Petticrew, M. (2021). Evaluation of public health interventions from a complex systems perspective: A research methods review. Social Science & Medicine, 272(113697), 113697. 10.1016/j.socscimed.2021.113697 33508655

[CIT0028] Michalowsky, B., Flessa, S., Eichler, T., Hertel, J., Dreier, A., Zwingmann, I., Wucherer, D., Rau, H., Thyrian, J. R., & Hoffmann, W. (2018). Healthcare utilization and costs in primary care patients with dementia: Baseline results of the DelpHi-trial. The European Journal of Health Economics, 19(1), 87–102. 10.1007/s10198-017-0869-7 28160100

[CIT0029] The Ministry of Health and Care Services. (2015). The Dementia Plan 2020. https://www.regjeringen.no/en/historical-archive/solbergs-government/andre-dokumenter/hod/2015/dementia-plan-2020/id2465117/

[CIT0030] Morrow-Howell, N., Halvorsen, C. J., Hovmand, P., Lee, C., & Ballard, E. (2017). Conceptualizing productive engagement in a system dynamics framework. Innovation in Aging, 1(1), igx018. 10.1093/geroni/igx018 30480112 PMC6177040

[CIT0031] Nakabe, T., Sasaki, N., Uematsu, H., Kunisawa, S., Wimo, A., & Imanaka, Y. (2019). Classification tree model of the personal economic burden of dementia care by related factors of both people with dementia and caregivers in Japan: A cross-sectional online survey. BMJ Open, 9(7), e026733. 10.1136/bmjopen-2018-026733 PMC6629423 31289069

[CIT0032] Nichols, E., Steinmetz, J. D., Vollset, S. E., Fukutaki, K., Chalek, J., Abd-Allah, F., Abdoli, A., Abualhasan, A., Abu-Gharbieh, E., & Akram, T. T. (2022). Estimation of the global prevalence of dementia in 2019 and forecasted prevalence in 2050: An analysis for the Global Burden of Disease Study 2019. Lancet Public Health, 7(2), e105–e125. 10.1016/S2468-2667(21)00249-8 34998485 PMC8810394

[CIT0033] The Norwegian Association for Public Health. (2019). https://nasjonalforeningen.no/

[CIT0034] Pinquart, M., & Sörensen, S. (2006). Helping caregivers of persons with dementia: Which interventions work and how large are their effects? International Psychogeriatrics, 18(4), 577–595. 10.1017/S1041610206003462 16686964

[CIT0035] Puaschitz, N. G., Jacobsen, F. F., Mannseth, J., Angeles, R. C., Berge, L. I., Gedde, M. H., & Husebo, B. S. (2021). Factors associated with access to assistive technology and telecare in home-dwelling people with dementia: Baseline data from the LIVE@ Home.Path trial. BMC Medical Informatics and Decision Making, 21(1), 264. 10.1186/s12911-021-01627-2 34525979 PMC8442311

[CIT0036] The Red Cross. (2019). Retrieved May 2023 from https://www.rodekors.no/

[CIT0037] Rodríguez‐González, A. M., Rodríguez‐Míguez, E., & Claveria, A. (2021). Determinants of caregiving burden among informal caregivers of adult care recipients with chronic illness. Journal of Clinical Nursing, 30(9-10), 1335–1346. 10.1111/jocn.15683 33528913

[CIT0038] Samus, Q. M., Johnston, D., Black, B. S., Hess, E., Lyman, C., Vavilikolanu, A., Pollutra, J., Leoutsakos, J. -M., Gitlin, L. N., Rabins, P. V., & Lyketsos, C. G. (2014). A multidimensional home-based care coordination intervention for elders with memory disorders: The maximizing independence at home (MIND) pilot randomized trial. The American Journal of Geriatric Psychiatry, 22(4), 398–414. 10.1016/j.jagp.2013.12.175 24502822 PMC4034346

[CIT0039] Schneider, L. S., & Olin, J. T. (1996). Clinical global impressions in Alzheimer’s clinical trials. International Psychogeriatrics, 8(2), 277–88; discussion 288. 10.1017/s1041610296002645 8994897

[CIT0040] Schulz, R., & Sherwood, P. R. (2008). Physical and mental health effects of family caregiving. American Journal of Nursing, 108((9 Suppl), 23–27; quiz 27. 10.1097/01.NAJ.0000336406.45248.4c PMC2791523 18797217

[CIT0041] Selbaek, G., Kirkevold, O., Sommer, O. H., & Engedal, K. (2008). The reliability and validity of the Norwegian version of the Neuropsychiatric Inventory, Nursing Home version (NPI-NH). International Psychogeriatrics, 20(2), 375–382. 10.1017/S1041610207005601 17559707

[CIT0042] Seys, D., Panella, M., VanZelm, R., Sermeus, W., Aeyels, D., Bruyneel, L., Coeckelberghs, E., & Vanhaecht, K. (2019). Care pathways are complex interventions in complex systems: New European Pathway Association framework. International Journal of Care Coordination, 22(1), 5–9. 10.1177/2053434519839195

[CIT0043] Skivington, K., Matthews, L., Simpson, S. A., Craig, P., Baird, J., Blazeby, J. M., Boyd, K. A., Craig, N., French, D. P., McIntosh, E., Petticrew, M., Rycroft-Malone, J., White, M., & Moore, L. (2021). A new framework for developing and evaluating complex interventions: Update of Medical Research Council guidance. British Medical Journal, 374:n2061. 10.1136/bmj.n2061 34593508 PMC8482308

[CIT0044] Stephan, A., Bieber, A., Hopper, L., Joyce, R., Irving, K., Zanetti, O., Portolani, E., Kerpershoek, L., Verhey, F., De Vugt, M., Wolfs, C., Eriksen, S., Røsvik, J., Marques, M. J., Gonçalves-Pereira, M., Sjölund, B. -M., Jelley, H., Woods, B., & Meyer, G.; Actifcare Consortium (2018). Barriers and facilitators to the access to and use of formal dementia care: Findings of a focus group study with people with dementia, informal carers and health and social care professionals in eight European countries. BMC Geriatrics, 18(1), 131. 10.1186/s12877-018-0816-1 29866102 PMC5987478

[CIT0045] Stroud, C., & Larson, E. B. (2021). Meeting the challenge of caring for persons living with dementia and their care partners and caregivers: A way forward. The National Academies Press. 10.17226/26026 33625814

[CIT0046] Tanner, J. A., Black, B. S., Johnston, D., Hess, E., Leoutsakos, J. -M., Gitlin, L. N., Rabins, P. V., Lyketsos, C. G., & Samus, Q. M. (2015). A randomized controlled trial of a community-based dementia care coordination intervention: Effects of MIND at Home on caregiver outcomes. American Journal of Geriatric Psychiatry, 23(4), 391–402. 10.1016/j.jagp.2014.08.002 PMC4355038 25260557

[CIT0047] Tooth, L., McKenna, K., Barnett, A., Prescott, C., & Murphy, S. (2005). Caregiver burden, time spent caring and health status in the first 12 months following stroke. Brain Injury, 19(12), 963–974. 10.1080/02699050500110785 16263639

[CIT0048] Van den Berg, B., & Spauwen, P. (2006). Measurement of informal care: An empirical study into the valid measurement of time spent on informal caregiving. Health Economics, 15(5), 447–460. 10.1002/hec.1075 16389664

[CIT0049] Velandia, P. P., Miller-Petrie, M. K., Chen, C., Chakrabarti, S., Chapin, A., Hay, S., Tsakalos, G., Wimo, A., & Dieleman, J. L. (2022). Global and regional spending on dementia care from 2000–2019 and expected future health spending scenarios from 2020–2050: An economic modelling exercise. EClinicalMedicine, 45, 101337. 10.1016/j.eclinm.2022.101337 35299657 PMC8921543

[CIT0050] Vislapuu, M., Angeles, R. C., Berge, L. I., Kjerstad, E., Gedde, M. H., & Husebo, B. S. (2021). The consequences of COVID-19 lockdown for formal and informal resource utilization among home-dwelling people with dementia: Results from the prospective PAN. DEM study. BMC Health Services Research, 21(1), 1–12. 10.1186/s12913-021-07041-8 34551783 PMC8457031

[CIT0051] Walter, E., & Pinquart, M. (2020). How effective are dementia caregiver interventions? An updated comprehensive meta-analysis. Gerontologist, 60(8), e609–e619. 10.1093/geront/gnz118 33226434

[CIT0052] Williams, F., Moghaddam, N., Ramsden, S., & De Boos, D. (2019). Interventions for reducing levels of burden amongst informal carers of persons with dementia in the community. A systematic review and meta-analysis of randomised controlled trials. Aging and Mental Health, 23(12), 1629–1642. 10.1080/13607863.2018.1515886 30450915

[CIT0053] Wimo, A., Gauthier, S., & Prince, M. (2018, July). Global estimates of informal care. *World Alzheimer report.* Alzheimer’s disease international (ADI) and Karolinska Institute. https://www.alzint.org/resource/global-estimates-of-informal-care/

[CIT0054] Wimo, A., Gustavsson, A., Jonsson, L., Winblad, B., Hsu, M. A., & Gannon, B. (2013). Application of Resource Utilization in Dementia (RUD) instrument in a global setting. Alzheimers & Dementia, 9(4), 429–435.e17. 10.1016/j.jalz.2012.06.008 23142433

[CIT0055] Wimo, A., Jonsson, L., & Zbrozek, A. (2010). The Resource Utilization in Dementia (RUD) instrument is valid for assessing informal care time in community-living patients with dementia. Journal of Nutrition, Health & Aging, 14(8), 685–690. 10.1007/s12603-010-0316-220922346

[CIT0056] Wimo, A., & Nordberg, G. (2007). Validity and reliability of assessments of time: comparisons of direct observations and estimates of time by the use of the resource utilization in dementia (RUD)-instrument. Archives of Gerontology & Geriatrics, 44(1), 71–81. 10.1016/j.archger.2006.03.001 16777246

[CIT0057] Wolford, B. (2019). *What is GDPR, the EU’s new data protection law?* https://gdpr-info.eu/

[CIT0058] World Medical Association. (2001). World medical association declaration of Helsinki. Ethical principles for medical research involving human subjects. Bulletin of the World Health Organization, 79(4), 373. https://www.ncbi.nlm.nih.gov/pmc/articles/PMC2566407/11357217 PMC2566407

[CIT0059] Wübker, A., Zwakhalen, S. M., Challis, D., Suhonen, R., Karlsson, S., Zabalegui, A., Soto, M., Saks, K., & Sauerland, D. (2015). Costs of care for people with dementia just before and after nursing home placement: Primary data from eight European countries. European Journal of Health Economics, 16(7), 689–707. 10.1007/s10198-014-0620-6 25069577

[CIT0060] Ydstebø, A. E., Benth, J., Bergh, S., Selbæk, G., & Vossius, C. (2020). Informal and formal care among persons with dementia immediately before nursing home admission. BMC Geriatrics, 20(1), 296. 10.1186/s12877-020-01703-8 32811440 PMC7436969

[CIT0061] You, E. C., Dunt, D., Doyle, C., & Hsueh, A. (2012). Effects of case management in community aged care on client and carer outcomes: A systematic review of randomized trials and comparative observational studies. BMC Health Services Research, 12(1), 1–14. 10.1186/1472-6963-12-395 23151143 PMC3508812

