# Supplementary material for: Feedback System Analysis of a Multicomponent Intervention on Dyads of Home-Dwelling Persons With Dementia and Their Caregivers: Results From the LIVE@Home.Path Trial
Source: Innov Aging. 2024 Feb 23;8(3):igae020. doi: 10.1093/geroni/igae020 (PMC10976912; doi:10.1093/geroni/igae020)
Supplement: igae020_suppl_Supplementary_Material [file igae020_suppl_supplementary_material.docx]

*Innovation in Aging* Supplementary Material: Vislapuu, Maarja; Patrascu, Monica; Allore, Heather; Husebo, Bettina Sandgathe; Kjerstad, Egil; Gedde, Marie Hidle; Berge, Line Iden. Feedback system analysis of a multicomponent intervention on dyads of home-dwelling persons with dementia and their caregivers. Results from the LIVE@Home.Path trial.

Section 1: additional details on the meaning and calculation of the *system error*.

In the design and modelling of feedback systems, the *system error* represents the *difference* between how the system should behave (the reference) and what the system actually does (its behavior as observable in the variation of the controlled variable). Depending on the type of systems, the *difference* is calculated in specific ways: the simplest is associated with common every-day feedback systems (e.g., a thermostat for regulating temperature in a room), while the most complex are the products of inference processes of evaluation (e.g., the decision of a human being acting as the regulatory system of the feedback loop). In this section, we present the calculation and meaning of the *system error* for these two examples.

**Example 1.A: System error calculation for a thermostat**

In room *P* (Figure 1.A), a thermostat reads via a sensor the ambient temperature *y(t)* over time as an outcome variable. Then, the controller *C* computes the difference between the current temperature and the desired reference value (objective) *r(t)* as the *system error*: *e(t) = r(t)*  ̶  *y(t)*. The error *e(t)* is processed by the decisional module of the controller *C_D_*, which then decides on the intervention (in system terminology called command) *u(t)*, that will perform the necessary action (e.g., adjusting the heaters to give more heat). When the system is affected by disturbances *d(t)* such as leaving the window open, the value of the *error* changes as well, which determines an adaptation of the intervention to the new context. The performances of the controller and the quality of the temperature in the room can be tracked via the variables *w(t)* and *v(t)*, respectively.

For example, assume the baseline temperature in the room is *y_t_* _= 0_ = 17⁰C. The desired reference temperature is *r_t_* _≥ 0_ = 22⁰C. This means that, at baseline (*t = 0*), the *error* is *e_t_* _= 0_ = 5⁰C. Now, assume that the heaters warm the room by 0.2⁰C every minute. Then, error at *t = 60* sec becomes *e_t_* _= 60_ = 4.8⁰C; at the next minute, it becomes *e_t_* _= 120_ = 4.6⁰C; this process continues until the error becomes approximately 0⁰C.

In feedback systems design, the baseline temperature can be generalized as “0” (undesired outcome) while the reference temperature can be generalized as “1” (desired outcome). As the feedback system evolves over time, the baseline outcome will increase toward the desired one. This concept can be applied to any feedback systems, from the simplest water heater to the most complex power plant.

**Figure 1.A: Thermostat with feedback.**

**
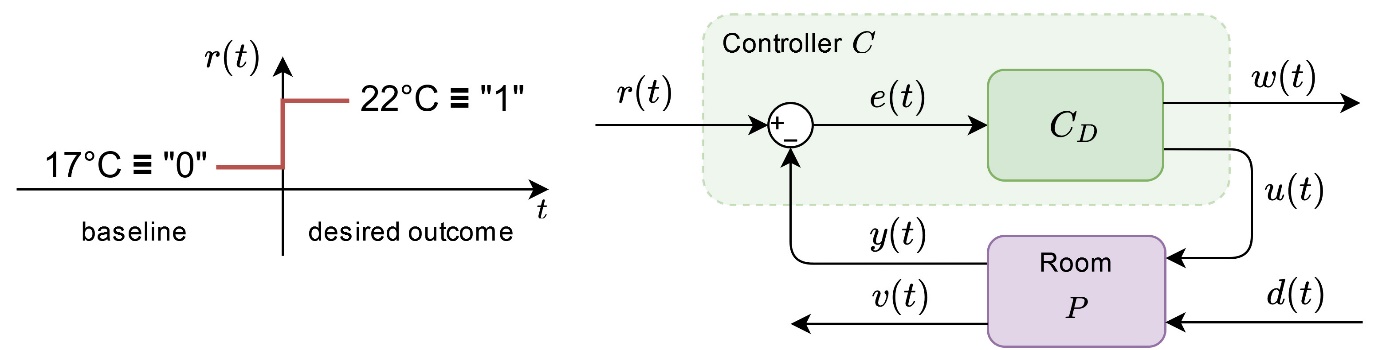
**

**Example 1.B: System error for the coordinator-dyad feedback configuration**

Extrapolating from Example 1.A, in the case of the coordinator-dyad feedback system, the dyad takes the place of the room, while the coordinator takes the place of the controller (Figure 1.B). The controlled variable *y_F_(t)* is represented by the information transmitted through follow-up from the dyad to the coordinator (the feedback). The reference is the objective *Obj(t)* that models the desired outcome for the dyad. The coordinator then *compares* the reference with the feedback, assessing whether the situation of the dyad is evolving toward the desired outcome. In this case, the comparison is the result of the inference process of the coordinator, taking into account various characteristics of the dyad’s status. The dyad is affected by disturbance *d(t)*, while the equivalent of the command is the multicomponent intervention *u_LIVE_(t)*.

To analyze the feedback system, we can abstract the reference as “0” for baseline and “1” as desired outcome. Numerically, the *system error* can also be abstracted as the difference between objective and follow-up: *e(t) = Obj(t)*  ̶  *y_F_(t)*. In this case, the resulting variable *e(t)* is a number between 0 and 1, but it is not a simple measure of, e.g., degrees, such as for temperature, but a quantification of how close to the objective the dyad has come (with the meaning of “0” closest and “1” farthest).

From the observer perspective of the researcher, variable *y_F_(t)* is not observable; instead, we quantify the status of the dyad through *v_CGIC-T,time_(t)* (i.e., the impression of change CGIC-T and the informal care *time* per RUD domain: ADL and IADL) and the performance of the coordinator through *w_CGIC-C_(t)* (coordinator impression of change). The research objectives concerning the dyad status and the time used for informal care are part of the general objective *Obj(t)* and thus, we can disambiguate these two dimensions of the reference to compare against the corresponding two dimensions of the follow-up, obtaining:

a) EG the general impression of change error: *EG(t)* = *Obj_CGIC-T_(t) ̶*  *v_CGIC-T_(t)*

b) ET_i∈{ADL,IADL}_ the time error: *ET_i_(t)* = *Obj_time_(t) ̶*  *v_time_(t)*

These two *system errors* are estimates of the error computed at the cognitive level of the coordinator for these outcome measures.

In this study, the objective dimensions *Obj_CGIC-T_(t)* and *Obj_time_(t)* are not numerically quantifiable (the coordinators did not aim to obtain a specific value for informal care time or CGIC-T). Therefore, in this paper we calculate the two errors using the equation of the sensitivity function for feedback systems and the Final Value Theorem (Åström & Murray, 2021).

**Figure 1.B: Coordinator-dyad feedback structure.**

**
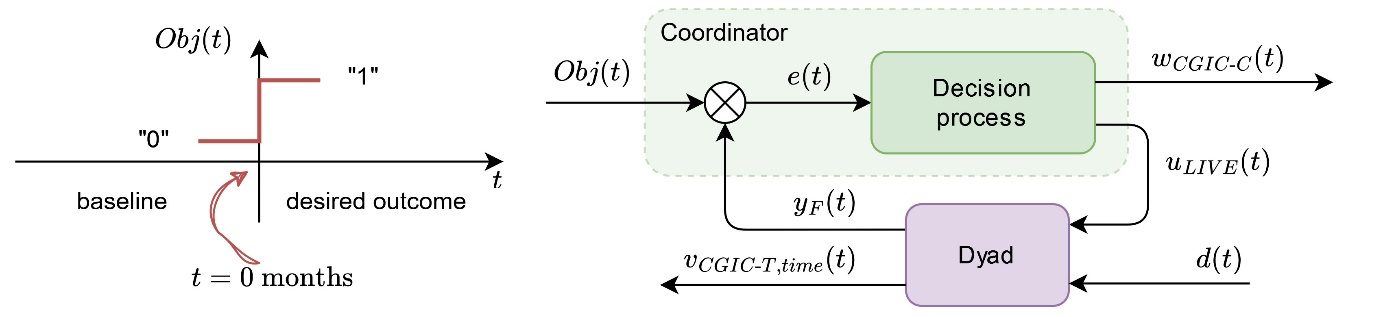
**

Section 2

Table 2.A: Baseline characteristics for people with dementia by attrition status during the first 6-month period of LIVE@Home.Path.

|  | Included  N=268 | Completers  (n=225) | Non-completers  (n=43) | P |
| --- | --- | --- | --- | --- |
| **Persons with dementia characteristics** |  |  |  |  |
| Age, mean (SD) | 82.2 (7.0) | 81.8 (6.9) | 84.5 (6.9) | **.019** |
| Gender, female, n (%) | 166 (62.2) | 141 (62.7) | 25 (58.1) | .700 |
| MMSE, mean (SD) | 20.7 (3.8) | 20.7 (3.8) | 20.4 (3.4) | .655 |
| NPI, median [IQR] | 13 [5; 25] | 12 [4; 24] | 16 [8; 29] | .051 |
| PSMS, mean (SD) | 10.4 (3.3) | 10.1 (3.0) | 11.7 (4.3) | **.011** |
| Informal care time, hours per day, mean (SD) |  |  |  |  |
| ADL | 1.5 (1.5) | 1.5 (1.5) | 1.6 (1.7) | .255 |
| IADL | 2.1 (1.8) | 2.1 (1.9) | 1.9 (1.5) | .844 |
| No. of municipality health care – or support services, median [IQR] | 1 [1; 2] | 1 [1; 2] | 2 [2; 3] | **<.001** |
| **Caregiver characteristics** |  |  |  |  |
| Age, mean (SD) | 65.9 (12.2) | 65.9 (12.2) | 65.8 (12.5) | .915 |
| Living with the caregivers, yes, n (%) | 120 (45.3) | 105 (46.7) | 15 (37.5) | .283 |
| Gender, female, n (%) | 174 (65.7) | 149 (66.2) | 25 (62.5) | .648 |
| Relationship, n (%) |  |  |  | .461 |
| - Spouse/Partner | 113 (42.6) | 98 (43.6) | 15 (37.5) |  |
| - Child | 138 (52.7) | 114 (50.7) | 24 (60.0) |  |
| - Other | 14 (5.3) | 13 (5.8) | 1 (2.5) |  |
| Education, n (%) |  |  |  | .246 |
| - Primary school | 15 (5.8) | 13 (5.8) | 2 (5.0) |  |
| - Secondary/vocational school | 75 (29.0) | 59 (26.2) | 16 (40.0) |  |
| - Higher education | 169 (65.3) | 147 (65.3) | 22 (55.0) |  |
| Working, yes, n (%) | 133 (49.6) | 112 (49.8) | 21 (52.5) | .853 |

**Legend:** N = total sample; n = number of patients; SD = standard deviation. Diff. between groups was tested with unequal variances t-test for normal and Wilcoxon-Mann-Whitney for non-normally distributed continuous variables and Pearson chi square tests for categorical variables. NPI = Neuropsychiatric Inventory sum of 12 items [range 0-144], higher scores indicate more frequent, severe and burdensome symptoms. PSMS = Physical Self-Maintenance Scale [range 0–30], higher score indicates lower functional capacity. Mini Mental Status Examination [range 0–30], a lower score indicates greater cognitive impairment, ADL = e.g., toileting, personal hygiene, and meal situations. IADL = e.g., taking medicine, out-patient visits.
